# Lipopolysaccharide Inhibits Alpha Epithelial Sodium Channel Expression via MiR-124-5p in Alveolar Type 2  Epithelial Cells

**DOI:** 10.1155/2020/8150780

**Published:** 2020-03-03

**Authors:** Yan Ding, Yong Cui, Zhiyu Zhou, Yapeng Hou, Xining Pang, Hongguang Nie

**Affiliations:** ^1^Department of Stem Cells and Regenerative Medicine, College of Basic Medical Science, China Medical University, Shenyang 110122, China; ^2^Department of Anesthesiology, First Affiliated Hospital of China Medical University, Shenyang 110001, China

## Abstract

Mesenchymal stem cells (MSCs) have been a potential strategy in the pretreatment of pulmonary diseases, while the mechanisms of MSCs-conditioned medium (MSCs-CM) involved with microRNAs on the regulation of lung ion transport are seldom reported. We investigated the role of miR-124-5p in lipopolysaccharide-involved epithelial sodium channel (ENaC) dysfunction and explored the potential target of miR-124-5p. We observed the lower expression of miR-124-5p after the administration of MSCs-CM, and the overexpression or inhibition of miR-124-5p regulated epithelial sodium channel *α*-subunit (*α*-ENaC) expression at protein levels in mouse alveolar type 2 epithelial (AT2) cells. We confirmed that *α*-ENaC is one of the target genes of miR-124-5p through dual luciferase assay and Ussing chamber assay revealed that miR-124-5p inhibited amiloride-sensitive currents associated with ENaC activity in intact H441 monolayers. Our results demonstrate that miR-124-5p can decrease the expression and function of *α*-ENaC in alveolar epithelial cells by targeting the 3′-UTR. The involvement of MSCs-CM in lipopolysaccharide-induced acute lung injury cell model could be related to the downregulation of miR-124-5p on *α*-ENaC, which may provide a new target for the treatment of acute lung injury.

## 1. Introduction

In addition to inflammation, acute lung injury/acute respiratory distress syndrome (ALI/ARDS) induces extensive capillary damage, leading to noncardiogenic pulmonary edema [1]. Therefore, the rate of alveolar fluid clearance (AFC) is a crucial prognostic factor for ALI/ARDS patients, which is mediated by ion transporters, including the alveolar epithelial sodium channel (ENaC) [2, 3]. ENaC is a heteromultimeric protein composed of *α*, *β*, *γ*, and *δ* subunits and is essential for the transepithelial absorption of Na^+^ and fluid from alveolar spaces [4, 5]. Among all the subunits, only *α*-ENaC presented Na^+^-dependent current consistent with active Na^+^ transport in *Xenopus laevis* oocytes, and in mice with a genetic deficiency for *α*-ENaC, the newborn were unable to clear airway fluid and died within 40 h of birth [6, 7]. These all suggest the critical importance of *α*-ENaC required for Na^+^ transport [8].

Short, noncoding RNAs termed microRNAs (miRs) bind predominantly to the untranslated regions (UTRs) of target mRNAs to decrease target protein expression [9, 10]. The involvement of miRs in ALI is still open to question. Previous studies suggested that miR-124-5p inhibited NF-*κ*B, cAMP/PKA, and MAPK/ERK pathways [11], all of which have been reported to be related to lipopolysaccharide- (LPS-) induced ALI [8, 12, 13]. Several bioinformatic websites such as TargetScan (http://www.targetscan.org/), PicTar (http://pictar. mdc-berlin.de/), and miRBase (http://www.mirbase. org/) were used to predict the targets of miR-124-5p. From this analysis, we found that *α*-ENaC is a potential target of miR-124-5p because the seed sequence of miR-124-5p aligns perfectly with the 3′-UTR of *α*-ENaC mRNA.

Here, we aimed to investigate whether miR-124-5p has biologic impacts on the alveolar epithelium, which may exert effects on the pathophysiology of ALI. Mesenchymal stem cells (MSCs) are a subset of cells with a variety of differentiation potential and previous studies have shown the benefits of MSCs-based therapy in respiratory injury [14]. Cell-free MSCs-conditioned medium (MSCs-CM) therapy has been demonstrated to confer pulmonary ischemic tolerance by paracrine factors, and we hypothesized that the administration of MSCs-CM would attenuate ALI through changing the delivery of miR-124-5p into epithelial cells, which will lead to the alteration in the protein expression of ENaC [15]. Using a combination of the LPS-induced ALI cell model, *in vitro* transfection assays, Western blot, and Ussing chamber, we demonstrated that MSCs-CM participates in the LPS-induced ALI cell model by alleviating the inhibition of miR-124-5p on the expression and function of *α*-ENaC. These findings support a potential therapeutic role for MSCs-CM in ALI through pleiotropic mechanisms.

## 2. Materials and Methods

### 2.1. Animals

All experiment methods involving C57 mice were performed according to the guidelines of the Animal Care and Use Committee, and all experimental protocols were approved by China Medical University. Mice were kept under pathogen-free conditions.

### 2.2. Preparation of Mouse MSCs-CM

Three-week-old, weighing 10–13 g pathogen-free C57 male mice were anaesthetized by diazepam (17.5 mg kg^−1^, intraperitoneally) followed by ketamine 6 min later (450 mg kg^−1^, intraperitoneally). The femora were isolated. Bone marrow was collected by gently washing medullary cavity of femora with DMEM/F12 medium (HyClone) supplemented with 10% fetal bovine serum (FBS, Gibco), 10 ng/ml recombinant mouse basic fibroblast growth factor (bFGF, PeproTech), 100 IU penicillin, and 100 *μ*g/ml streptomycin. Then, it was cultured in a humidified incubator (5% CO_2_, 37°C) for 24 h. The medium was first changed to remove nonadherent cells and tissues and then replaced every other day. MSCs were passaged after 80% confluent and the cells of the 2^nd^ and 3^rd^ passages were used to collect MSCs-CM. At 80% confluence, the medium of MSCs was changed and replaced by FBS-deprived DMEM/F12 medium. MSCs-CM was collected in 24 h and stored at −80°C freezer after filtering with a 0.22 *μ*m filter.

### 2.3. Isolation and Culture of Mouse Alveolar Type 2 Epithelial Cells

Isolated lungs from newborn mice (within 24 h) were separated by lobes in cold phosphate buffer saline (PBS). Teased lung tissue was digested with trypsin (Sigma) and collagenase (Sigma) for 30 min, respectively. Cells were filtrated and cultured in 5% CO_2_, 37°C atmosphere in DMEM/F12 containing 10% FBS (Gibco), 100 IU penicillin, and 100 *μ*g/ml streptomycin for 45 min. Unattached cells were collected and the above culture process was repeated 4 times to remove lung fibroblast cells. Then, the cell suspension was transferred on the IgG coated culture dish and incubated for 30 min to remove lymphocytes, macrophages, and neutrophils. Unattached cells were adjusted to 2-3 × 10^6^/ml and the medium was changed after 72 h for the first time and then changed every other day.

### 2.4. Culture of H441 Cells and H441 Monolayers

Human distal lung epithelial cell line NCI-H441 was obtained from the American Type Culture Collection (ATCC) and cultured as previously described [16]. H441 cells were grown in RPMI medium (ATCC) containing 10% FBS, 2 mM L-glutamine, 10 mM HEPES, 1 mM sodium pyruvate, 4.5 g/L glucose, 1.5 g/L sodium bicarbonate, and antibiotics (100 U/ml penicillin and 100 *μ*g/ml streptomycin). For Ussing chamber assays, cells were seeded on permeable support filters (Costar) at a supraconfluent density (∼5 × 10^6^ cells/cm^2^) and incubated in a humidified atmosphere of 5% CO_2_ at 37°C. Dexamethasone (250 nM, Sigma) was supplemented to stimulate ENaC expression. Cells reached confluency in the Costar Transwells 24 h after plating. At this point, media and nonadherent cells in the apical compartment were removed to adapt the cells to the air-liquid interface culture. Culture media in the basolateral compartment were replaced every other day, whereas the apical surface was rinsed with PBS. Transepithelial resistance was measured by an epithelial tissue volt-ohm-meter (World Precision Instruments). Highly polarized tight monolayers with resistance >500 Ω cm^2^ were used for measuring short-circuit current (*I*_*sc*_) levels of transepithelium.

### 2.5. Ussing Chamber Assay

Measurements of transepithelial *I*_*sc*_ and resistance were performed as described previously in H441 monolayers [17]. In brief, H441 monolayers were mounted in Ussing chambers (Physiologic Instrument) and then bathed on both sides with a solution containing (in mM): 120 NaCl, 25 NaHCO_3_, 3.3 KH_2_PO_4_, 0.83 K_2_HPO_4_, 1.2 CaCl_2_, 1.2 MgCl_2_, 10 HEPES sodium salt, and either 10 mannitol for apical compartment or 10 D-glucose for basolateral compartment. The pH of each solution was adjusted to 7.4 and the osmolality was 290–300 mOsm/kg. The solutions of both sides were bubbled with mixed gas containing 95% O_2_ and 5% CO_2_ at 37°C. *I*_*sc*_ was measured with Ag-AgCl electrodes filled with 4% agar in 3 M KCl. The monolayers were short-circuited to 0 mV, and a 10 mV pulse of 1 s was applied every 10 s to monitor the resistance of transepithelium. Acquire and Analyze 2.3 program was used to collect data. ENaC activity is the reduction of *I*_*sc*_ after adding amiloride to the apical side (100 *μ*M, amiloride-sensitive *I*_*sc*_).

### 2.6. Western Blot Analysis

Mouse alveolar type 2 epithelial (AT2) cells were cultured in 6-well plates until 80% confluence and washed three times with PBS and then harvested for immunoblot assays. Proteins were separated on 10% SDS-PAGE gels and transferred onto PVDF membranes (Invitrogen). The blots were incubated in blocking solution containing 20 mM Tris-HCl (pH 7.5), 0.5 M NaCl, 1% Tween (TBST), and 5% BSA for 1 h at room temperature. Then, the membranes were incubated with primary antibody: *α*-ENaC antibody (PA1-920A, Thermo Fisher) and *β*-actin (sc-47778, Santa Cruz Biotechnology) at 1 : 2000 in TBST containing 5% BSA at 4°C overnight. The specific band between 70 and 100 kDa for *α*-ENaC protein could be seen, according to the manufacturer's manual and previous studies [18]. Following washing three times with TBST, membranes were incubated with HRP conjugated goat-anti-rabbit or goat-anti-mouse secondary antibody at 1 : 2000 at room temperature for 1 h and then washed for 10 min with TBST three times. Images were developed by an enhanced chemiluminescence (ECL) kit and collected by the ImageJ program.

### 2.7. Real-Time Polymerase Chain Reaction

Total RNA was extracted using TRIzol reagent (Invitrogen) according to the manufacturer's instructions, and spectrophotometry was used to measure the concentration and purity of total RNA. Reverse transcription was performed using the PrimeScript RT reagent Kit with gDNA Eraser (TaKaRa) and real-time polymerase chain reaction (RT-PCR) with SYBR Premix Ex Taq (TaKaRa) was performed using LightCycler 480 System. The MiR-124-5p level was measured using the Mir-X miRNA First-Strand Synthesis Kit (TaKaRa) and U6 was used as a reference. The following primer sequences were used: miR-124-5p (5′-CGT GTT CAC AGC GGA CCT TGA T-3′), U6 forward (5′-GGA ACG ATA CAG AGA AGA TTA GC-3′) and reverse (5′-TGG AAC GCT TCA CGA ATT TGC G-3′). The data from RT-PCR were analyzed using the 2^−△△CT^ method.

### 2.8. Transfections

The negative control, miR-124-5p mimics, miRNA inhibitor negative control, or miR-124-5p inhibitor (GenePharma) was transfected into cells with siRNA-mate (GenePharma) according to the manufacturer's instructions. The final concentration of miR-124-5p mimics was 30 nM and the miR-124-5p inhibitor was 60 nM. All transfection reagents were removed after 6 h and cells were used 48 h after transfection.

### 2.9. Dual Luciferase Reporter Assay

The luciferase reporter assay was performed to test whether miR-124-5p directly binds to the 3′-UTR of *α*-ENaC mRNA. The luciferase reporter plasmid, pmirGLO-*α*-ENaC 3′-UTR, was purchased from GenePharma. Each fragment was of two types: wild-type and mutant. Cotransfection of AT2 cells with the dual luciferase reporter plasmids and miR-124-5p mimics or negative controls were transfected into cells with siRNA-mate (GenePharma). After 48 h, luciferase activity was measured using the Dual Luciferase Reporter Assay Kit (Vazyme), according to the manufacturer's instructions.

### 2.10. Statistical Analysis

All results were presented as mean ± SE. ENaC activity is the difference in the total and amiloride-resistant current fractions. Normality and homoscedasticity test was done by Levene and Shapiro–Wilk test before applying parametric tests. For the comparison of the two groups, we used Student's two-tailed *t*-test; for the comparison of multiple groups, we performed one-way analysis of variance (ANOVA) followed by Bonferroni's test for all the groups of the experiment. When the data did not pass the normality or homoscedasticity test, we used a nonparametric *t*-test (Mann–Whitney *U* test). Variations were considered significant when the *P* value was less than 0.05. Statistical analysis was performed with Origin 8.0.

## 3. Results

### 3.1. MSCs-CM Decreases the Expression of MiR-124-5p in Mouse AT2 Cells

**Figure 1:**
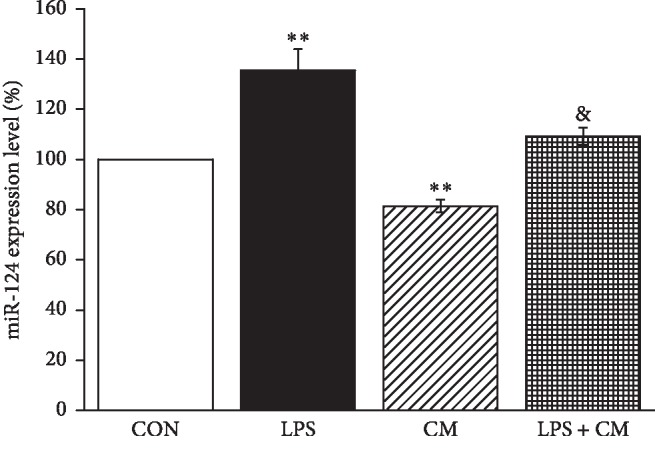
MiR-124-5p is increased in the LPS-induced ALI cell model. The result of real-time PCR assays shows miR-124-5p level in control AT2 cells (CON), LPS (10 *μ*g/ml, 12 h)-treated AT2 cells (LPS), MSCs-CM (24 h-treated CON group) (CM), and MSCs-CM (24 h-treated LPS group) (LPS + CM). The relative level of miR-124-5p was calculated as miR-124-5p/U6 ratios. ^*∗∗*^*P* < 0.01, compared with the CON group; ^&^*P* < 0.05, compared with the LPS group; *N* = 4.

Previous studies suggested that miR-124-5p involved in the corresponding pathways is related to LPS-induced ALI [11]. In our experiment, we first analyzed the levels of miR-124-5p in mouse AT2 cells after LPS and MSCs-CM administration. We found that exposure to LPS caused a significant increase in miR-124-5p level compared with control which was set as 100% (*P* < 0.01, *n* = 4) (Figure 1). Conversely, the levels of miR-124-5p decreased in normal and LPS-treated mouse AT2 cells after the administration of MSCs-CM (*P* < 0.01 versus control group, and *P* < 0.05 versus LPS group, *n* = 4), respectively. These data proved that the expression level of miR-124-5p increased during ALI and MSCs-CM downregulated the level of miR-124-5p in the LPS-induced ALI cell model.

### 3.2. The Level of MiR-124-5p Is Negatively Correlated with *α*-ENaC in Mouse AT2 Cells

**Figure 2:**
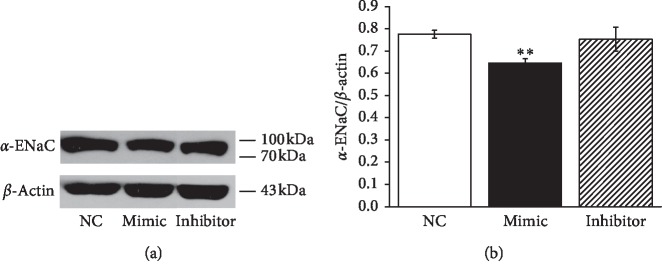
The levels of miR-124-5p and *α*-ENaC are negatively correlated in mouse AT2 cells. (a) Representative Western blot measurement of *α*-ENaC protein expression in AT2 cells transfected with miR-124-5p negative control (NC), miR-124-5p mimic (Mimic), and miR-124-5p inhibitor (Inhibitor) for 48 h. (b) Graphical representation of data obtained from Western blots and quantified through gray analysis (*α*-ENaC/*β*-actin). ^*∗∗*^*P* < 0.01, compared with the NC group; *N* = 4.

Previous studies demonstrated that LPS downregulated *α*-ENaC mRNA expression [19], and we further predicted the binding site between miR-124-5p and *α*-ENaC. Accordingly, we assumed that LPS may inhibit the expression of *α*-ENaC protein through miR-124-5p. To test this hypothesis, mouse AT2 cells were transfected with miR-124-5p mimics (Mimic) or inhibitor (Inhibitor), respectively. The effect of miR-124-5p on *α*-ENaC was examined by Western blot analysis (Figures 2(a) and 2(b)). Transfection of miR-124-5p mimics into mouse AT2 cells resulted in a significant decrease of *α*-ENaC expression compared with the negative control (NC) group (*P* < 0.01, *n* = 4), while the inhibition of miR-124-5p showed no difference (*n* = 4). These data suggested that miR-124-5p suppressed the protein expression of *α*-ENaC. The potential mechanism of ALI induced by LPS may be related to the enhanced miR-124-5p level and sequent inhibition of *α*-ENaC protein expression.

### 3.3. MiR-124-5p Downregulates *α*-ENaC Expression by Binding to the 3′-UTR

**Figure 3:**
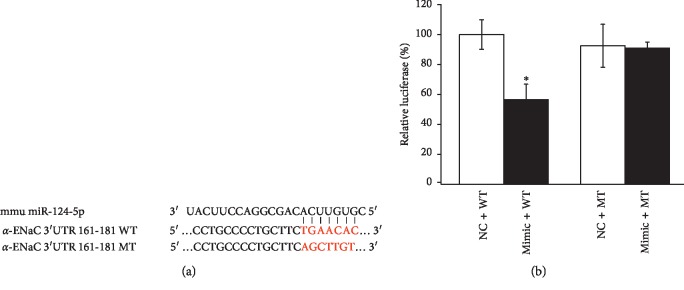
MiR-124-5p downregulates *α*-ENaC expression by binding to the 3′-untranslated region (3′-UTR). (a) The prediction of the miR-124-5p binding site located in *α*-ENaC mRNA. Alignment of miR-124-5p with wild-type (WT) and mutant (MT) *α*-ENaC mRNA 3′-UTR. (b) Dual luciferase assay for AT2 cells cotransfected with miR-124-5p negative control (NC) or miR-124-5p mimic (Mimic) together with pmirGLO-*α*-ENaC (WT or MT) for 24 h ^*∗*^*P* < 0.05, compared with NC group; *N* = 3.

Potential miR-124-5p targets were predicted using in silico approaches, and according to the bioinformatic website prediction, we estimated that *α*-ENaC is a potential target of miR-124-5p. To confirm this finding, a dual luciferase target gene assay was conducted. Wild-type and mutant reporter gene vectors for *α*-ENaC were constructed, and the sequences of 3′-UTRs of the wild-type and mutant *α*-ENaC were shown in Figure 3(a). Mouse AT2 cells were cotransfected with the vectors (NC) and miR-124-5p mimics (Mimic). After 24 h, the *fireﬂy* luciferase activity was measured. *Fireﬂy* luciferase units were normalized with *Renilla* luciferase units. We found that the expression of pmirGLO-*α*-ENaC-WT (Mimic + WT) relative luciferase activity was reduced to 56.53 ± 9.92% by miR-124-5p (*P* < 0.05, compared with NC + WT), while the expression of pmirGLO-*α*-ENaC-MT (Mimic + MT) was not suppressed significantly by miR-124-5p (*P* > 0.05, compared with NC + MT, Figure 3(b)). The above data verified that *α*-ENaC is one of the target genes of miR-124-5p.

### 3.4. MiR-124-5p Inhibits Amiloride-Sensitive Short-Circuit Current in H441 Monolayers

**Figure 4:**
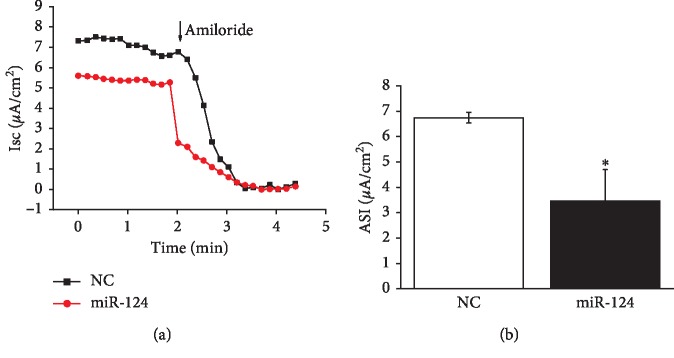
MiR-124-5p inhibits amiloride-sensitive short-circuit current in H441 monolayers. (a) Representative short-circuit current (*I*_*sc*_) traces recorded in confluent H441 monolayers transfected with miR-124-5p negative control (NC) and miR-124-5p mimic (miR-124) for 48 h. (b) Average amiloride-sensitive *I*_*sc*_ (ASI) in NC and miR-124 group. ^*∗*^*P* < 0.05, compared with the NC group; *N* = 3.

Human bronchoalveolar epithelial-derived Clara (H441) cells have been extensively applied in studying the function of ENaC in the lung, and ENaC properties of H441 are similar to those of primary AT2 cells, which could hardly grow into monolayers [20, 21]. Amiloride-sensitive AFC reflects ENaC-mediated fluid transport. To further confirm the regulation of miR-124-5p on ENaC activity, we measured *I*_*sc*_ in confluent H441 monolayers. As shown in Figure 4(a), amiloride-sensitive *I*_*sc*_ (ASI) was defined as the difference between the total current and the amiloride-resistant current, and miR-124-5p decreased ASI from 6.74 ± 0.21 to 3.45 ± 1.24 (*μ*A/cm^2^) (*P* < 0.05 versus control, *n* = 3, Figure 4(b)). These data indicated that miR-124-5p could inhibit the ion transport of lung through decreasing ENaC activity in alveolar epithelial cells.

### 3.5. MSCs-CM and MiR-124-5p Inhibitor Increase the Protein Expression of *α*-ENaC in AT2 Cells after LPS Administration

**Figure 5:**
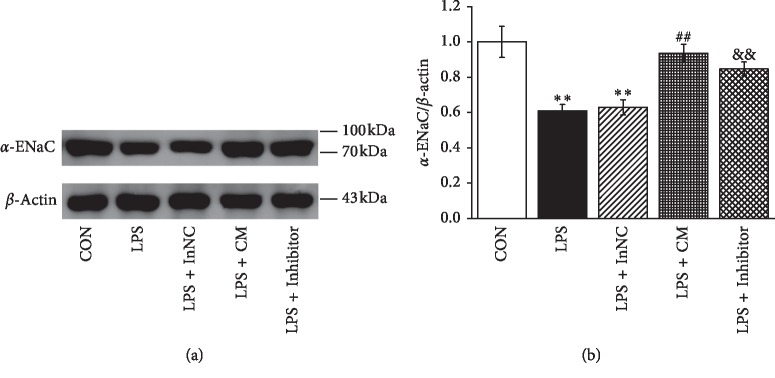
MSCs-CM and miR-124-5p inhibitor increase the protein expression of *α*-ENaC in AT2 cells after LPS administration. (a) Representative Western blot measurement of *α*-ENaC protein expression in control AT2 cells (CON), LPS (10 *μ*g/ml, 12 h)-treated AT2 cells (LPS), MSCs-CM (24 h treated LPS group) (LPS + CM), LPS-treated AT2 cells transfected with miR-124-5p inhibitor negative control (LPS + InNC), or miR-124-5p inhibitor (LPS + Inhibitor) for 48 h. (b) Graphical representation of data obtained from Western blots and quantified through gray analysis (*α*-ENaC/*β*-actin). ^*∗∗*^*P* < 0.01, compared with the CON group; ^##^*P* < 0.01, compared with the LPS group; ^&&^*P* < 0.01, compared with LPS + InNC group; *N* = 4.

To further test our hypothesis that MSCs-CM may exert beneficial effects in ALI by inhibiting the LPS-enhanced miR-124-5p expression in AT2 cells, LPS-treated AT2 cells were administrated with MSCs-CM or transfected with miR-124-5p inhibitor negative control (LPS + InNC) or miR-124-5p inhibitor (LPS + Inhibitor), respectively. The *α*-ENaC expression was examined by Western blot analysis (Figures 5(a) and 5(b)). LPS downregulated *α*-ENaC expression in AT2 cells compared with the control (CON) group (*P* < 0.01, *n* = 4). Incubating with MSCs-CM caused a significant increase of *α*-ENaC expression compared with LPS alone (*P* < 0.01, *n* = 4), and inhibition of miR-124-5p caused a similar increase of *α*-ENaC expression compared with inhibitor negative control group in LPS-treated AT2 cells (*P* < 0.01, *n* = 4). These data suggested that the potential mechanism of MSCs-CM therapy for ALI induced by LPS may be related to reducing the miR-124-5p level and subsequently increasing *α*-ENaC protein expression.

### 3.6. MSCs-CM and MiR-124-5p Inhibitor Enhance Amiloride-Sensitive Short-Circuit Currents in H441 Monolayers after LPS Administration

**Figure 6:**
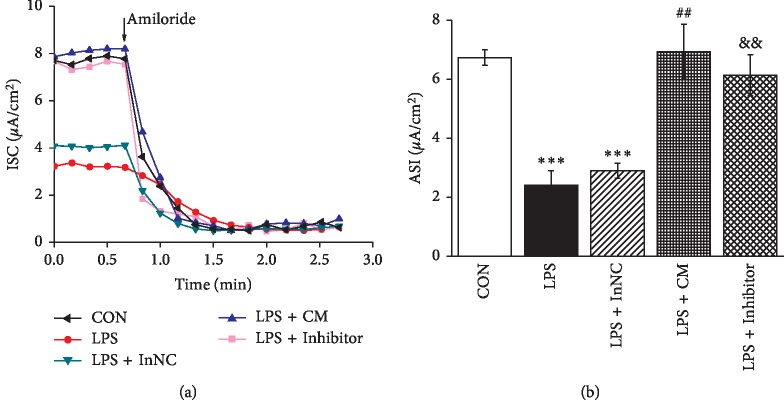
MSCs-CM and miR-124-5p inhibitor enhance amiloride-sensitive short-circuit currents in H441 monolayers after LPS administration. (a) Representative short-circuit current (*I*_*sc*_) traces recorded in confluent H441 monolayers (CON), LPS (10 *μ*g/ml, 12 h)-treated H441 cells (LPS), MSCs-CM (24 h treated LPS group) (LPS + CM), LPS-treated H441 cells transfected with miR-124-5p inhibitor negative control (LPS + InNC), and miR-124-5p inhibitor (LPS + Inhibitor) for 48 h. (b) Average amiloride-sensitive *I*_*sc*_ (ASI) in groups. ^*∗∗∗*^*P* < 0.001, compared with the CON group; ^##^*P* < 0.01, compared with the LPS group; ^&&^*P* < 0.01, compared with LPS + InNC group; *N* = 3.

To test if the above regulation mechanism of MSCs-CM is also related to the function of ENaC, we measured *I*_*sc*_ in confluent H441 monolayers in the above groups. As shown in Figures 6(a) and 6(b), LPS decreased ASI from 6.73 ± 0.27 to 2.40 ± 0.50 (*μ*A/cm^2^) (*P* < 0.001 versus control, *n* = 3), indicating that LPS could inhibit the ion transport of lung through decreasing ENaC activity in alveolar epithelial cells. Incubating with MSCs-CM caused a significant increase of ENaC activity compared with LPS-treated AT2 cells (*P* < 0.01, *n* = 3), and inhibition of miR-124-5p caused a similar increase of ENaC activity compared with inhibitor negative control group in LPS-treated AT2 cells (*P* < 0.01, *n* = 3). These data showed that, compared with LPS alone, the ASI reflecting ENaC activity increased after the transfection of miR-124-5p inhibitor or administration with MSCs-CM in AT2 cells, which nearly recovered to the state of the control group.

## 4. Discussion

Pulmonary edema is a major characteristic of LPS-induced ALI/ARDS, which is associated with a leak of fluid into the lung interstitium and the alveolar space. Recovery from ALI requires the removal of fluid from the alveolar space, which contributes to the development, severity, and outcome of pulmonary edema in humans [22]. The AFC process is crucial to efficient gas exchange in the lung, and patients with ALI that have intact AFC have lower morbidity and mortality than those with compromised AFC [23, 24].

The active transport of Na^+^ by AT2 cells provides a major driving force for the removal of fluid from the alveolar space. Previous studies revealed that AFC strongly depends on the function of ENaC on the apical surface and Na^+^/K^+^-ATPase on the basolateral surface of alveolar epithelial cells [25]. There were studies revealing that the upregulation of ENaC improved edema fluid reabsorption during pulmonary edema after thiourea-induced lung injury [26]. Because of its role in the regulation of Na^+^ homeostasis, ENaC has been associated with clinical defects of Na^+^ and water transport [27]. Among the four ENaC subunits, altogether three are expressed in mice, named *α-*, *β-*, and *γ*-ENaC. Among *αβγ*-ENaC subunits, the *α*-subunit is believed to form electrically detectable Na^+^-selective channels, whereas the *β*- and *γ*-subunits serve as accessory regulatory subunits that can increase, by two orders of magnitude, the amplitude of channel activity from the *α*-subunit alone [16, 28, 29]. In our previous studies, LPS could reduce the expression of *α-* and *γ*-ENaC, and we have found that MSCs-CM has similar effects on *γ*-ENaC. Based on several bioinformatic websites, we have predicted that *α*-ENaC is a potential target of miR-124-5p, for the seed sequence of miR-124-5p aligns perfectly with the 3′-UTR of *α*-ENaC mRNA. Accordingly, we chose the *α*-ENaC subunit as our target for in-depth study.

**Figure 7:**
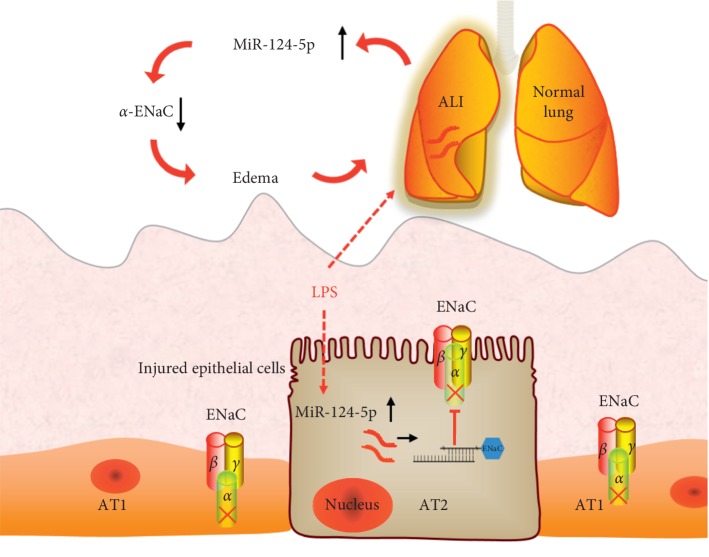
Potential mechanisms for miR-124-5p involved in LPS-induced lung epithelial injury cell model. The level of miR-124-5p increases during LPS-induced ALI, which can bind 3′-UTR of *α*-ENaC and decrease its expression and function in alveolar epithelial cells, leading to the occurrence of pulmonary edema. ALI: acute lung injury; ENaC: epithelial sodium channel; *Α*Τ1: alveolar type 1 epithelial cells; *Α*Τ2: alveolar type 2 epithelial cells.

The miRs are an extensive family of short (approximately 22 nucleotides), noncoding RNAs. They function as posttranscriptional repressors of gene expression by binding predominantly to the 3′-UTR of mRNAs to interfere with protein production [30, 31]. There is currently little information about miRs and Na^+^ regulation [32]. MiR-124-5p has been reported to be related to lung epithelial maturation, but the mechanisms of MSCs-CM involved with miRs on the regulation of lung ion transport are seldom reported [11]. In this study, we first found that the expression of miR-124-5p in mouse AT2 cells decreased after the administration of MSCs-CM, contrary to the effects of LPS alone. A combination of MSCs-CM and LPS could reverse the LPS-induced enhancement of miR-124-5p expression. Consequently, the overexpression or inhibition of miR-124-5p demonstrated the regulation of *α*-ENaC at the protein level. Western blot analysis showed a marked decrease of *α*-ENaC protein expression in mouse AT2 cells transfected by miR-124-5p. Next, we confirmed that *α*-ENaC is one of the target genes of miR-124-5p by Luciferase reporter assay. To test the effects of miR-124-5p on ion transport function, *I*_*sc*_ was measured by Ussing chamber assay, and we revealed that miR-124-5p inhibited amiloride-sensitive currents associated with ENaC activity in intact H441 monolayers. Finally, the protein expressions of *α*-ENaC and ENaC activity were performed, and as expected, both MSCs-CM and miR-124-5p inhibitor caused a significant increase of ENaC expression and activity, which offset the effects of LPS alone. Taken together, our data showed that the level of miR-124-5p increased in the LPS-induced ALI cell model, which could bind 3′-UTR of *α*-ENaC mRNA and led to the reduction of its expression and function in alveolar epithelial cells, causing the formation of pulmonary edema (Figure 7). Meanwhile, MSCs-CM could increase ENaC expression and activity through reducing the miR-124-5p level to treat LPS-induced ALI.

For recording *I*_*sc*_, the data from primary AT2 cells could be more convincing, but it is a pity that primary AT2 cells from mice are hard to form tight monolayers in the air-liquid culture mode and it is nearly impossible to record the *I*_*sc*_ in these cells for the technique difficulties [33, 34]. H441 is human Clara-like airway epithelial cells, which can easily form monolayers at the air-liquid interface and exhibit a number of recognized morphological structures and functional similarities to primary human AT2 cells [20, 21, 35, 36]. Therefore, we recorded the *I*_*sc*_ in H441 monolayers in our electrophysiological experiment but applied the other experiments in mouse AT2 cells.

MSCs have been a potential strategy in the pretreatment of pulmonary diseases and MSCs-CM may be a preferable therapeutic option for ALI [37]. MiR-124-3p transferred by MSCs-derived exosomes had been demonstrated to inhibit purinergic receptor P2X ligand-gated ion channel 7P2X7 expression, thus improving oxidative stress injury and suppressing inflammatory response in traumatic ALI [38, 39]. In our experiment, we focused on miR-124-5p and found the inhibition of miR-124-5p on *α*-ENaC by binding the 3′-UTR. MSCs-CM may contain some paracrine factors and inhibit miR-124-5p accordingly by affecting the microenvironment in AT2 cells, and the potential mechanism of MSCs-CM therapy for LPS-induced ALI may be related to reducing the miR-124-5p level and subsequently increasing ENaC protein expression and activity, which benefits the resolution of edema in ALI. However, how the expression level of miR-124-5p was downregulated in MSCs-CM was still a direction that awaits our further investigation.

## 5. Conclusions

We demonstrate that MSCs-CM may involve in the LPS-induced ALI cell model by the regulation of miR-124-5p, which can decrease the expression and activity of *α*-ENaC in alveolar epithelial cells through targeting the 3′-UTR. This is a novel mechanism for MSCs-CM and may provide a new target for the treatment of ALI.

## Data Availability

The data used to support the findings of this study are available from the corresponding author upon reasonable request.

## Supplementary Materials

Supplementary Materials Supplementary Figure 1: the full-length blots/gels of *α*-ENaC protein extracted from AT2 cells treated with MSCs-CM for 24 h. MSCs-CM were collected from MSCs transfected with miR-124-5p negative control (NC), miR-124-5p mimic (Mimic), miR-124-5p inhibitor negative control (In + NC), and miR-124-5p inhibitor (Inhibitor) for 48 h. Molecular weights of all bands have been indicated on the right of the blot. The specific band between 70 and 100 kDa for *α*-ENaC protein could be seen, according to the manufacturer's manual.

## References

[1] Qi D., He J., Wang D. (2014). 17*β*-estradiol suppresses lipopolysaccharide-induced acute lung injury through PI3K/Akt/SGK1 mediated up-regulation of epithelial sodium channel (ENaC) in vivo and in vitro. *Respiratory Research*.

[2] Matthay M. A. (2014). Resolution of pulmonary edema. Thirty years of progress. *American Journal of Respiratory and Critical Care Medicine*.

[3] Shen C. H., Lin J. Y., Chang Y. L. (2018). Inhibition of NKCC1 modulates alveolar fluid clearance and inflammation in ischemia-reperfusion lung injury via TRAF6-mediated pathways. *Frontiers in Immunology*.

[4] Ji H.-L., Zhao R.-Z., Chen Z.-X., Shetty S., Idell S., Matalon S. (2012). *δ* ENaC: a novel divergent amiloride-inhibitable sodium channel. *American Journal of Physiology-Lung Cellular and Molecular Physiology*.

[5] Matalon S., Bartoszewski R., Collawn J. F. (2015). Role of epithelial sodium channels in the regulation of lung fluid homeostasis. *American Journal of Physiology-Lung Cellular and Molecular Physiology*.

[6] Canessa C. M., Horisberger J.-D., Rossier B. C. (1993). Epithelial sodium channel related to proteins involved in neurodegeneration. *Nature*.

[7] Hummler E., Barker P., Gatzy J. (1996). Early death due to defective neonatal lung liquid clearance in *α*ENaC-deficient mice. *Nature Genetics*.

[8] Deng J., Wang D.-x., Liang A.-l., Tang J., Xiang D.-k. (2017). Effects of baicalin on alveolar fluid clearance and *α*-ENaC expression in rats with LPS-induced acute lung injury. *Canadian Journal of Physiology and Pharmacology*.

[9] Lewis B. P., Shih I.-h., Jones-Rhoades M. W., Bartel D. P., Burge C. B. (2003). Prediction of mammalian microRNA targets. *Cell*.

[10] Guo H., Ingolia N. T., Weissman J. S., Bartel D. P. (2010). Mammalian microRNAs predominantly act to decrease target mRNA levels. *Nature*.

[11] Wang Y., Huang C., Chintagari N. R., Xi D., Weng T., Liu L. (2015). miR-124 regulates fetal pulmonary epithelial cell maturation. *American Journal of Physiology-Lung Cellular and Molecular Physiology*.

[12] Lee J.-p., Li Y.-c., Chen H.-y. (2010). Protective effects of luteolin against lipopolysaccharide-induced acute lung injury involves inhibition of MEK/ERK and PI3K/Akt pathways in neutrophils. *Acta Pharmacologica Sinica*.

[13] Xia H., Wang J., Sun S. (2019). Resolvin D1 alleviates ventilator-induced lung injury in mice by activating PPAR*γ*/NF-*κ*B signaling pathway. *BioMed Research International*.

[14] Khatri M., Richardson L. A. (2017). Therapeutic potential of porcine bronchoalveolar fluid-derived mesenchymal stromal cells in a pig model of LPS-induced ALI. *Journal of Cellular Physiology*.

[15] Hwang B., Liles W. C., Waworuntu R., Mulligan M. S. (2016). Pretreatment with bone marrow-derived mesenchymal stromal cell-conditioned media confers pulmonary ischemic tolerance. *The Journal of Thoracic and Cardiovascular Surgery*.

[16] Cui Y., Li H., Wu S. (2016). Formaldehyde impairs transepithelial sodium transport. *Scientific Reports*.

[17] Chen L., Patel R. P., Teng X., Bosworth C. A., Lancaster J. R., Matalon S. (2006). Mechanisms of cystic fibrosis transmembrane conductance regulator activation by S-nitrosoglutathione. *Journal of Biological Chemistry*.

[18] Rahman M. S., Gandhi S., Otulakowski G., Duan W., Sarangapani A., O’Brodovich H. (2010). Long-term terbutaline exposure stimulates *α*1-Na+-K+-ATPase expression at posttranscriptional level in rat fetal distal lung epithelial cells. *American Journal of Physiology-Lung Cellular and Molecular Physiology*.

[19] Migneault F., Boncoeur É., Morneau F., Pascariu M., Dagenais A., Berthiaume Y. (2013). Cycloheximide and lipopolysaccharide downregulate *α*ENaC mRNA via different mechanisms in alveolar epithelial cells. *American Journal of Physiology-Lung Cellular and Molecular Physiology*.

[20] Nie H. G., Chen L., Han D. Y. (2009). Regulation of epithelial sodium channels by cGMP/PKGII. *The Journal of Physiology*.

[21] Nie H., Cui Y., Wu S., Ding Y., Li Y. (2016). 1,25-Dihydroxyvitamin D enhances alveolar fluid clearance by upregulating the expression of epithelial sodium channels. *Journal of Pharmaceutical Sciences*.

[22] Deng W., Li C. Y., Tong J. (2019). Insulin ameliorates pulmonary edema through the upregulation of epithelial sodium channel via the PI3K/SGK1 pathway in mice with lipopolysaccharideinduced lung injury. *Molecular Medicine Reports*.

[23] Hummler E., Planès C. (2010). Importance of ENaC-mediated sodium transport in alveolar fluid clearance using genetically-engineered mice. *Cellular Physiology and Biochemistry*.

[24] Ware L. B., Matthay M. A. (2001). Alveolar fluid clearance is impaired in the majority of patients with acute lung injury and the acute respiratory distress syndrome. *American Journal of Respiratory and Critical Care Medicine*.

[25] Niu F., Xu X., Zhang R., Sun L., Gan N., Wang A. (2019). Ursodeoxycholic acid stimulates alveolar fluid clearance in LPS-induced pulmonary edema via ALX/cAMP/PI3K pathway. *Journal of Cellular Physiology*.

[26] Egli M., Duplain H., Lepori M. (2004). Defective respiratory amiloride-sensitive sodium transport predisposes to pulmonary oedema and delays its resolution in mice. *The Journal of Physiology*.

[27] Edinger R. S., Coronnello C., Bodnar A. J. (2014). Aldosterone regulates microRNAs in the cortical collecting duct to alter sodium transport. *Journal of the American Society of Nephrology*.

[28] Chang S. S., Grunder S., Hanukoglu A. (1996). Mutations in subunits of the epithelial sodium channel cause salt wasting with hyperkalaemic acidosis, pseudohypoaldosteronism type 1. *Nature Genetics*.

[29] Kellenberger S., Schild L. (2015). International union of basic and clinical pharmacology. XCI. Structure, function, and pharmacology of acid-sensing ion channels and the epithelial Na+ channel. *Pharmacological Reviews*.

[30] Bartel D. P. (2009). MicroRNAs: target recognition and regulatory functions. *Cell*.

[31] Flynt A. S., Lai E. C. (2008). Biological principles of microRNA-mediated regulation: shared themes amid diversity. *Nature Reviews Genetics*.

[32] Zhou Y., Li P., Goodwin A. J. (2019). Exosomes from endothelial progenitor cells improve outcomes of the lipopolysaccharide-induced acute lung injury. *Critical Care*.

[33] Demaio L., Tseng W., Balverde Z. (2009). Characterization of mouse alveolar epithelial cell monolayers. *American Journal of Physiology-Lung Cellular and Molecular Physiology*.

[34] Tzotzos S., Fischer B., Fischer H. (2013). AP301, a synthetic peptide mimicking the lectin-like domain of TNF, enhances amiloride-sensitive Na+ current in primary dog, pig and rat alveolar type II cells. *Pulmonary Pharmacology & Therapeutics*.

[35] Korbmacher J. P., Michel C., Neubauer D. (2014). Amiloride-sensitive fluid resorption in NCI-H441 lung epithelia depends on an apical Cl^−^ conductance. *Physiological Reports*.

[36] Albert A. P., Woollhead A. M., Mace O. J., Baines D. L. (2008). AICAR decreases the activity of two distinct amiloride-sensitive Na+-permeable channels in H441 human lung epithelial cell monolayers. *American Journal of Physiology-Lung Cellular and Molecular Physiology*.

[37] Goolaerts A., Pellan-Randrianarison N., Larghero J. (2014). Conditioned media from mesenchymal stromal cells restore sodium transport and preserve epithelial permeability in an in vitro model of acute alveolar injury. *American Journal of Physiology-Lung Cellular and Molecular Physiology*.

[38] Li Q.-C., Liang Y., Su Z.-B. (2019). Prophylactic treatment with MSC-derived exosomes attenuates traumatic acute lung injury in rats. *American Journal of Physiology-Lung Cellular and Molecular Physiology*.

[39] Aliotta J. M., Pereira M., Wen S. (2016). Exosomes induce and reverse monocrotaline-induced pulmonary hypertension in mice. *Cardiovascular Research*.

