# Supplementary material for: Lipopolysaccharide Inhibits Alpha Epithelial Sodium Channel Expression via MiR-124-5p in Alveolar Type 2 Epithelial Cells
Source: Biomed Res Int. 2020 Mar 3;2020:8150780. doi: 10.1155/2020/8150780 (PMC7072113; doi:10.1155/2020/8150780)

**Mesenchymal Stem Cells-Conditioned Medium Involves Acute Lung Injury through MiR-124-5p Targeting Alpha Epithelial Sodium Channel**

**Yan Ding,^1^ Yong Cui,^2^ Zhiyu Zhou,^1^ Yapeng Hou,^1^ Xining Pang,^1^ Hongguang Nie^1^**

*^1^Department of Stem Cells and Regenerative Medicine, College of Basic Medical Science, China Medical University, Shenyang, 110122, China*

*^2^Department of Anesthesiology, First Affiliated Hospital of China Medical University, Shenyang, 110001, China*

Correspondence should be addressed to Hongguang Nie; [hgnie@cmu.edu.cn](mailto:hgnie@cmu.edu.cn)

Supplementary Figure 1. The full-length blots/gels of α-ENaC protein extracted from AT2 cells treated with MSCs-CM for 24 h. MSCs-CM were collected from MSCs transfected with miR-124-5p negative control (NC), miR-124-5p mimic (Mimic), miR-124-5p inhibitor negative control (In + NC) and miR-124-5p inhibitor (Inhibitor) for 48 h. Molecular weights of all bands have been indicated on the right of the blot. The specific band between 70 and 100 kDa for α-ENaC protein could be seen, according to the manufacturer’s manual.

Supplementary Figure 1


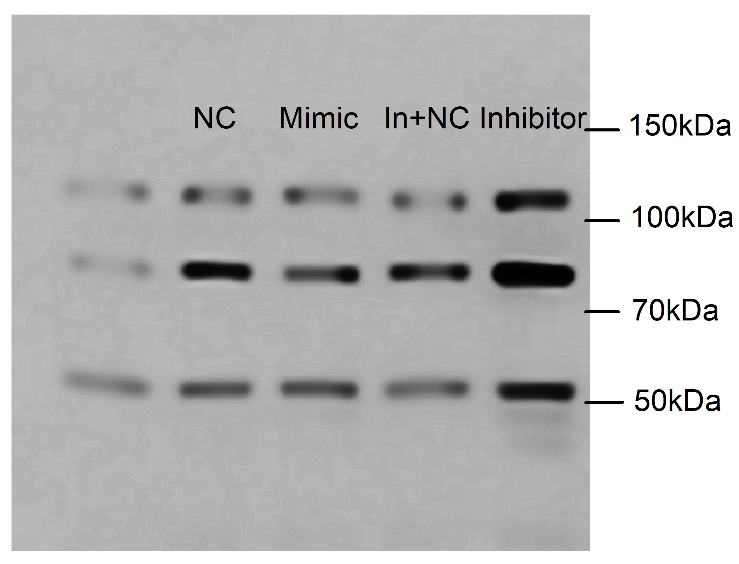

Supplement: Supplementary Materials — Supplementary Figure 1: the full-length blots/gels of α-ENaC protein extracted from AT2 cells treated with MSCs-CM for 24 h. MSCs-CM were collected from MSCs transfected with miR-124-5p negative control (NC), miR-124-5p mimic (Mimic), miR-124-5p inhibitor negative control (In + NC), and miR-124-5p inhibitor (Inhibitor) for 48 h. Molecular weights of all bands have been indicated on the right of the blot. The specific band between 70 and 100 kDa for α-ENaC protein could be seen, according to the manufacturer's manual. [file 8150780.f1.doc]
